# Epidemiology and Outcomes of Hospitalizations Due to Hepatocellular Carcinoma

**DOI:** 10.7759/cureus.20089

**Published:** 2021-12-01

**Authors:** Sanjana Mullangi, Praneeth R Keesari, Anas Zaher, Yashwitha Sai Pulakurthi, Frank Adusei Poku, Arathi Rajeev, Prasanna Lakshmi Vidiyala, Asha Latha Guntupalli, Maheshkumar Desai, Jessica Ohemeng-Dapaah, Yaw Asare, Achint A Patel, Manidhar Lekkala

**Affiliations:** 1 Internal Medicine, Hillcrest Medical Center, Tulsa, USA; 2 Internal Medicine, Kamineni Academy of Medical Sciences and Research Center, Hyderabad, IND; 3 Internal Medicine, University of Debrecen, Debrecen, HUN; 4 Internal Medicine, University of Central Oklahoma, Edmond, USA; 5 Internal Medicine, Government Medical College Kozhikode, Kozhikode, IND; 6 Internal Medicine, New Vision University, Tbilisi, GEO; 7 Internal Medicine, NRI Medical College and General Hospital, Chinna Kakani, IND; 8 Internal Medicine, Hamilton Medical Center, Medical College of Georgia/Augusta University, Dalton, USA; 9 Internal Medicine, Trust Hospital, Accra, GHA; 10 Epidemiology and Public Health, School of Public Health, University of Ghana, Accra, GHA; 11 Internal Medicine, Oak Hill Hospital, Brooksville, USA; 12 Oncology, University of Kansas Medical Center, Kansas City, USA

**Keywords:** trends, outcome, in-hospital mortality, hepatocellular carcinoma (hcc), hepatocellular carcinoma

## Abstract

Background

Hepatocellular Carcinoma (HCC) is a severe complication of cirrhosis and the incidence of HCC has been increasing in the United States (US). We aim to describe the trends, characteristics, and outcomes of hospitalizations due to HCC across the last decade.

Methods

We derived a study cohort from the Nationwide Inpatient Sample (NIS) for the years 2008-2017. Adult hospitalizations due to HCC were identified using the International Classification of Diseases (9th/10th Editions) Clinical Modification diagnosis codes (ICD-9-CM/ICD-10-CM). Comorbidities were also identified by ICD-9/10-CM codes and Elixhauser Comorbidity Software (Agency for Healthcare Research and Quality, Rockville, Maryland, US). Our primary outcomes were in-hospital mortality and discharge to the facility. We then utilized the Cochran-Armitage trend test and multivariable survey logistic regression models to analyze the trends, outcomes, and predictors.

Results

A total of 155,436 adult hospitalizations occurred due to HCC from 2008-2017. The number of hospitalizations with HCC decreased from 16,754 in 2008 to 14,715 in 2017. Additionally, trends of in-hospital mortality declined over the study period but discharge to facilities remained stable. Furthermore, in multivariable regression analysis, predictors of increased mortality in HCC patients were advanced age (OR 1.1; 95%CI 1.0-1.2; p< 0.0001), African American (OR 1.3; 95%CI 1.1-1.4;p< 0.001), Rural/ non-teaching hospitals (OR 2.7; 95%CI 2.4-3.3; p< 0.001), uninsured (OR 1.9; CI 1.6-2.2; p< 0.0001) and complications like septicemia and pneumonia as well as comorbidities such as hypertension, diabetes mellitus, and renal failure. We observed similar trends in discharge to facilities.

Conclusions

In this nationally representative study, we observed a decrease in hospitalizations of patients with HCC along with in-hospital mortality; however, discharge to facilities remained stable over the last decade. We also identified multiple predictors significantly associated with increased mortality, some of which are potentially modifiable and can be points of interest for future studies.

## Introduction

Hepatocellular carcinoma (HCC) is the most prevalent primary liver cancer and one of the leading causes of cancer-related deaths [1]. In the United States (US), HCC causes significant morbidity and mortality, as the incidence and mortality rates have increased over the past decades [2,3]. HCC constitutes 2.2% of all new cancer cases and 5% of all cancer deaths, with a five-year relative survival of 20.3% [4]. Per the current statistics, the incidence and mortality rates of HCC in the US are projected to increase significantly by 2030 [2].

The majority of HCC develops from underlying liver cirrhosis or chronic liver inflammation, commonly from hepatitis B or C virus (HBV or HCV) infection and heavy alcohol consumption [1]. The increase in incidence and mortality from HCC is attributable to longstanding HCV infection and advancing age in US adults born between 1945 and 1965 [5]. However, changes in dietary patterns, increased prevalence of obesity, nonalcoholic fatty liver disease, socioeconomic status, age structure, and migration have resulted in a shift in the epidemiology and etiology of HCC in the US [6,7]. For example, universal HBV vaccination, the use of direct-acting antivirals to treat HCV, and the influx of migrants from HBV-endemic countries have contributed to these changes [8,9]. Moreover, comprehensive screening for HCC and effective treatment for HBV and HCV can potentially decrease HCC incidence and hospitalization rates. These risk factors significantly affect the incidence, hospitalization, and mortality rates for HCC. Previous population-based studies that utilized data from different databases in the US showed that between 2008 and 2015, the incidence of HCC either plateaued or remained stable [10-12]. The impact of this on HCC hospitalizations during the aforementioned and the years after is unknown. The American Association for the Study of Liver Diseases recommended biannual screening and surveillance for HCC in high-risk patients such as those with cirrhosis in 2005 [13]. There is some fair evidence to suggest that screening leads to early detection of HCC, improved curative rates, and improved survival [14]. However, this screening and surveillance have been inconsistent [15,16]. The extent to which screening for HCC impacts the trends in diagnosis, incidence, and mortality remains to be elucidated.

Over the past couple of decades, advances in therapeutic approaches including ablation therapy, liver resection, transarterial chemoembolization (TACE), and liver transplantation, have become available for patients [1]. Since 2007, systemic therapy with agents such as Sorafenib for advanced HCC has demonstrated improved survival by several months [17,18]. Indeed, prior studies have demonstrated a downward trend in the in-hospital mortality associated with HCC between 2002 and 2014 [19-21]. However, these studies do not reflect the current trends for HCC hospitalizations, length of stay (LOS), and outcomes. Therefore, we sought to update the trends in HCC hospitalizations, LOS, in-hospital mortality and examine the predictors of mortality from 2008 through 2017 in the US using the National Inpatient Sample (NIS) database. The results of this study can help in the assessment of healthcare needs and the planning of healthcare services for patients and families affected by HCC.

## Materials and methods

Data source

We extracted our study cohort from the NIS database of the Healthcare Cost and Utilization Project (HCUP), Agency for Healthcare Research and Quality (AHRQ ) [22]. NIS is one of the largest all-payer publicly available databases on inpatient discharges from US hospitals maintained by the AHRQ [22]. The NIS approximates a 20% stratified sample of discharges from US community hospitals, excluding rehabilitation and long-term acute care hospitals, and contains more than seven million hospitalizations annually [22]. With the established weights in NIS, this data could be weighted to represent the standardized US hospital inpatient population and obtain national estimates with high accuracy [23]. The NIS is released every year, and this allows the analysis of trends over time [5,19,24-26]. At present, 47 states including the District of Columbia contribute data to the NIS.

Study population and design

We queried the 2008-2017 NIS database using International Classification of Diseases, 9th Revision, Clinical Modification and International Classification of Diseases, 10th Revision, Clinical Modification (ICD-9/10-CM) diagnose codes for HCC. These codes have been used by previously published studies that utilized administrative databases such as the NIS [5,19,24]. The inclusion criteria were limited to hospitalizations with HCC as the primary diagnosis. We extracted demographics, hospital-level characteristics (geographical region, size, and teaching status), and patient-level characteristics as supplied as part of NIS [27]. We estimated comorbidities using Elixhauser Comorbidity Software, Version 3.7 (HCUP, AHRQ, Rockville, Maryland, US) and mortality risk using the validated All Patient Refined Diagnosis Related Groups (APR-DRG) severity score (3M™ APR-DRG Software, 3M Company, Maplewood, Minnesota, US) [28-30]. Specific concurrent medical conditions and procedures of interest were identified by ICD-9/10-CM diagnosis and procedure codes.

Statistical analysis

Descriptive statistics were performed to present the baseline sociodemographic, comorbidities, and hospital-level characteristics of HCC hospitalizations. Proportions and means were used to summarize categorical and continuous variables, respectively. The exposure variable was the calendar year of HCC hospitalizations. The outcomes of interest were trends in HCC hospitalizations, in-hospital mortality, discharge to facility (skilled nursing facility (SNF), intermediate care facility, short-term hospital), LOS, and predictors of poor outcomes (in-hospital mortality and discharge to facility). Baseline characteristics of study cohort were expressed in proportions (%) for categorical variables and as mean (standard error) plus median (interquartile range) for numerical variables. Trend of hospitalizations due to HCC was expressed as raw numbers of hospitalizations and discharge disposition was expressed as a proportion of HCC hospitalizations for each year. Poor outcome was defined as in-hospital mortality or discharge to facilities. Among adult patients, discharge to facility such as SNF is associated with higher mortality and readmissions [31,32]. To identify the predictors of poor outcomes (in-hospital mortality and discharge to facility), we performed multivariable logistic regression with covariates that included demographic and hospital-level characteristics, and multiple comorbid conditions, and the results are presented as OR with 95% CI. For the regression analysis, survey procedures were used to account for the inherent survey design of NIS to produce more robust estimates [33]. We utilized SAS® 9.3 software (©2011, SAS Institute Inc., Cary, North Carolina, US) for all analyses and included designated weight values to produce nationally representative estimates [23]. A two-tailed p-value <0.05 was considered statistically significant for all analyses.

## Results

**Figure 1:**
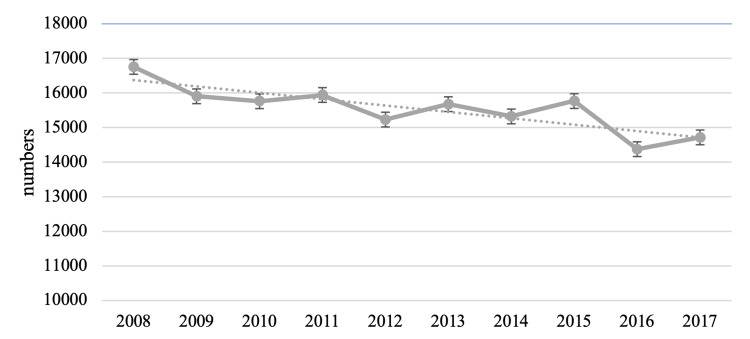
Temporal trends of hospitalizations due to HCC HCC: hepatocellular carcinoma

There were 155,435 hospitalizations due to HCC among adults aged ≥18 years in the US between 2008 and 2017. The absolute number of HCC hospitalizations decreased from 16,754 (10.8) in 2008 to 14,715 in 2017 (9.4%) (P=0.001) (Figure 1).

Baseline characteristics of hospitalizations due to HCC

The baseline characteristics of persons hospitalized for HCC within the study period are outlined in Table 1. In summary, most hospitalizations (91.5%) with HCC were adults aged >50 years with a median age of 62 (IQR, 55-70) years, 73.3% were males, 53.9% were Caucasians, and 63% had Medicare/Medicaid. The associated co-morbidities included liver diseases (63%), hypertension (52.5%), diabetes mellitus (31.6%), alcoholism (17.2%), and obesity (7.1%). Most of the HCC hospitalizations were in large bed-sized (69.9%) and urban teaching hospitals (74%).

**Table 1:** Demographic and co-morbid characteristics of hospitalizations due to hepatocellular carcinoma in the United States, 2008-2017

Characteristics	Hospitalizations
Overall	155436
Age in years (mean±SE)	63.34 (0.1)
Age in years (median [q1-q3])	62 (55-70)
Age in years (%)	
18-34	1.7
35-49	6.7
50-64	48.3
65-79	33.5
>=80	9.7
Gender (%)	
Male	73.4
Female	26.6
Race (%)	
White	54.0
Black	16.5
Hispanic	15.1
Others	14.5
Comorbidities (%)	
Obesity	7.1
Hypertension	52.5
Diabetes mellitus with chronic complications	6.1
diabetes mellitus without chronic complications	25.5
Congestive heart failure	6.1
Valvular heart disease	1.5
History of chronic pulmonary disease	14.2
Pulmonary circulatory disease	2.5
Peripheral vascular disease	4.2
Paralysis	0.9
Coagulopathy	20.2
solid tumor without metastasis	1.1
lymphoma	0.5
Metastatic cancer	3.3
Weightloss	13.5
Liver disease	63.0
Alcoholism	17.2
other neurological disorders	4.5
renal failure	10.3
hypothyroidism	7.4
arthritis	1.2
anemia deficiency	22.2
fluid and electrolyte disorders	35.0
depression	7.5
psychoses	2.8
Drug abuse	5.0
AIDS	0.9
Peptic ulcer disease	0.4
Median house hold income (%)	
1st quartile	31.4
2nd quartile	24.8
3rd quartile	23.1
4th quartile	20.7
Primary Insurance (%)	
Medicare/Medicaid	63.1
Private including HMO	27.2
Uninsured/Self-pay	9.8
Hospital bed size (%)	
Small	9.4
Medium	21.0
Large	69.6
Hospital Type (%)	
Rural	4.1
Urban-Non teaching	21.6
Teaching	74.3
Hospital region (%)	
Northeast	22.7
Midwest	17.3
South	36.5
West	23.5
Day of admission	
weekday	83.7
weekend	16.3
Source of admission (%)	
Transfer from other hospital or other health facility	51.4
Emergency department	48.7
Type of admission (%)	
Emergent or Urgent	68.4
Elective	31.6

Discharge disposition of hospitalizations due to HCC

**Figure 2:**
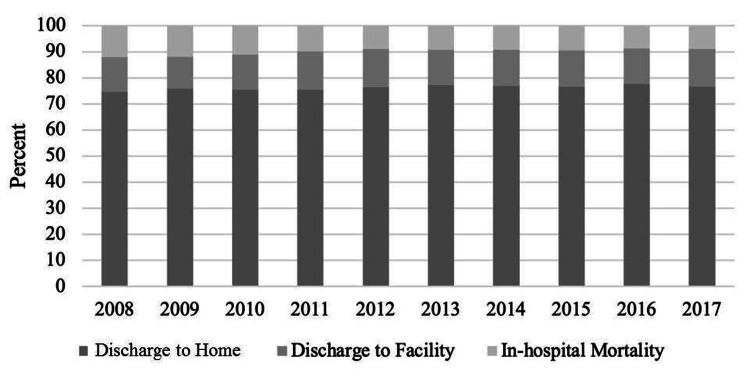
Temporal trends of discharge disposition of hospitalizations due to HCC HCC: hepatocellular carcinoma

Figure 2 demonstrates the trends of in-hospital mortality among the HCC hospitalizations. In-hospital mortality significantly declined from 12.1% in 2008 to 8.9% in 2017 (P<0.001). The proportion of HCC patients discharged to home experienced a steady rise over the years from 75% in 2008 to 78% in 2017 (P=0.009). There was no significant change over time in the proportion of HCC patients discharged to facility (13.3% in 2008 to 14.3% in 2017 (P=0.15)). The mean LOS of hospitalized patients was 6 ± 1 day. The median LOS was four (IQR, 2-7) days.

Predictors of in-hospital mortality and discharge to facilities among hospitalizations due to HCC

In multivariable logistic regression analysis (Table 2), increased odds of mortality were associated with male gender (OR 1.27; 95% CI: 1.51-1.4; P< 0.0001), each 10-year increment in age (OR 1.2; 95% CI: 1.1-1.2; P<0.0001), Black race (OR 1.3; 95%CI: 1.16-1.46; P<0.0001), and no health insurance (OR 1.91; 95% CI: 1.65 - 2.22, P<0.001).

**Table 2:** Predictors of in-hospital mortality among hospitalizations due to HCC LL: lower limit; UL: upper limit; HCC: hepatocellular carcinoma

Independent variable/Characteristic	Odd Ratio	95% CI (LL)	95% CI (UL)	P value
Age (10 years increase)	1.15	1.10	1.20	< 0.001
Gender (%)				
Male	1.27	1.15	1.40	< 0.001
Female	ref			
Race (%)				
White	ref			
Black	1.30	1.16	1.46	< 0.001
Hispanic	1.06	0.93	1.20	0.4157
Others	1.04	0.92	1.19	0.5240
Comorbidities (%)				
Obesity	0.65	0.53	0.79	< 0.001
Hypertension	0.61	0.56	0.67	< 0.001
Diabetes mellitus	0.79	0.71	0.88	< 0.001
Congestive heart failure	1.16	0.98	1.37	0.0922
Hospital onset Pneumonia	1.29	1.06	1.56	0.0102
Septicemia	6.03	5.18	7.01	< 0.001
History of chronic pulmonary disease	0.93	0.82	1.06	0.2822
Pulmonary circulatory disease	1.59	1.26	1.99	< 0.001
Chronic Hypertension	0.61	0.56	0.67	< 0.001
Peripheral vascular disease	1.07	0.88	1.30	0.4944
Neurological disease	1.47	1.23	1.76	< 0.001
Paralysis	1.18	0.79	1.75	0.4210
Coagulopathy	1.36	1.23	1.51	< 0.001
Metastatic cancer	0.76	0.59	0.97	0.0298
Weightloss	1.00	0.89	1.13	0.9872
Electrolytes	2.06	1.88	2.26	< 0.001
Liver disease	1.00	0.91	1.09	0.9508
Alcoholism	0.76	0.68	0.86	< 0.001
Renal failure	1.32	1.15	1.51	< 0.001
Hypothyrodism	0.71	0.59	0.85	0.0002
Psyciatric diseases	0.63	0.47	0.85	0.0028
Depression	0.80	0.67	0.95	0.0128
Median house hold income (%)				
1st quartile	1.14	0.99	1.30	0.0627
2nd quartile	1.02	0.89	1.16	0.8086
3rd quartile	1.04	0.92	1.18	0.5546
4th quartile	ref			
Primary Insurance (%)				
Medicare/Medicaid	ref			
Private including HMO	1.54	1.37	1.73	< 0.001
Uninsured/Self-pay	1.91	1.65	2.22	< 0.001
Hospital bed size (%)				
Small	1.74	1.48	2.04	< 0.001
Medium	1.24	1.10	1.39	0.0003
Large	ref			
Hospital Type (%)				
Rural	2.72	2.24	3.30	< 0.001
Urban-Non teaching	1.58	1.41	1.77	< 0.001
Teaching	ref			
Hospital region (%)				
Northeast	ref			
Midwest	0.77	0.65	0.92	0.0030
South	0.77	0.67	0.90	0.0006
West	0.93	0.79	1.09	0.3707

Mortality was not significantly different among household income quartiles. Comorbidities that were associated with increased odds of mortality included sepsis (OR 6.03; 95% CI: 5.18 - 7.01, P<0.001), pneumonia (OR 1.29; 95% CI: 1.06-1.56, P=0.01), pulmonary circulatory disorders (OR 1.59; 95% CI: 1.26 - 1.99; P<0.0001), renal failure, coagulopathy, and electrolyte disorders. In terms of hospital level characteristics, admission to rural (OR 2.72; 95% CI: 2.24 - 3.30; P<0.0001), urban non-teaching hospitals (OR 1.58; 95% CI: 1.41 - 1.77; P<0.0001), as well as small (OR 1.74; 95% CI: 1.48 - 2.04; P<0.0001), and medium (OR 1.24; 95% CI: 1.10 - 1.39; P<0.0001) bed size hospitals.

Factors that were significantly associated with increased odds of discharge to facility included increasing age (OR 1.34; 95% CI:1.3 - 1.4; P<0.001) and admission in rural (OR 2.66; 95% CI: 2.24 -3.16; P<0.0001) or urban non-teaching hospitals (OR 1.9; 95% CI: 1.73 - 2.10; P<0.0001). Comorbidities that increased the odds of discharge to facility were paralysis (OR 2.63; 95% CI: 1.93 - 3.60; P<0.0001), septicemia (OR 2.55; 95% CI: 2.08 - 3.14; P<0.0001), psychiatric diseases (OR 2.11; 95% CI: 1.74 - 2.54; P<0.0001), neurologic disease (OR 1.9; 95% CI: 1.64 - 2.20; P<0.0001) and others (Table 3).

**Table 3:** Predictors of discharge disposition to facility among hospitalizations due to HCC LL: lower limit; UL: upper limit; HCC: hepatocellular carcinoma

Independent variable/Characteristic	Odd Ratio	95% CI (LL)	95% CI (UL)	P value
Age (10 years increase)	1.34	1.29	1.40	< 0.001
Gender (%)				
Male	ref			
Female	1.03	0.95	1.12	0.5071
Race (%)				
White	ref			
Black	1.11	0.99	1.24	0.0708
Hispanic	0.79	0.69	0.90	0.0003
Others	0.78	0.69	0.89	0.0002
Comorbidities (%)				
Obesity	1.05	0.91	1.21	0.5274
Hypertension	0.86	0.79	0.93	< 0.001
Diabetes mellitus	0.91	0.83	0.99	0.0250
Congestive heart failure	1.37	1.19	1.57	< 0.001
Hospital onset Pneumonia	1.63	1.37	1.94	< 0.001
Septicemia	2.55	2.08	3.14	< 0.001
History of chronic pulmonary disease	1.04	0.94	1.15	0.4048
Pulmonary circulatory disease	0.96	0.81	1.14	0.6227
Chronic Hypertension	0.86	0.79	0.93	< 0.001
Peripheral vascular disease	1.28	1.03	1.58	0.0284
Neurologic disease	1.90	1.64	2.20	< 0.001
Paralysis	2.63	1.93	3.60	< 0.001
Coagulopathy	1.20	1.09	1.31	< 0.001
Metastatic cancer	1.02	0.83	1.26	0.8587
Weightloss	1.55	1.41	1.72	< 0.001
Electrolytes	1.99	1.84	2.15	< 0.001
Liver disease	1.04	0.95	1.13	0.4047
Alcoholism	1.11	1.00	1.23	0.0461
Renal failure	1.29	1.15	1.44	< 0.001
Hypothyroidism	0.98	0.86	1.12	0.7256
Psychiatric diseases	2.11	1.74	2.54	< 0.001
Depression	1.40	1.24	1.59	< 0.001
Median house hold income (%)				
1st quartile	1.13	1.01	1.27	0.0402
2nd quartile	1.09	0.97	1.22	0.1722
3rd quartile	1.14	1.02	1.28	0.0224
4th quartile				
Primary Insurance (%)				
Medicare/Medicaid				
Private including HMO	0.68	0.62	0.76	< 0.001
Uninsured/Self-pay	0.79	0.68	0.92	0.0026
Hospital bed size (%)				
Small	1.59	1.39	1.81	< 0.001
Medium	1.38	1.25	1.53	< 0.001
Large				
Hospital Type (%)				
Rural	2.66	2.24	3.16	< 0.001
Urban-Non teaching	1.90	1.73	2.10	< 0.001
Teaching				
Hospital region (%)				
Northeast				
Midwest	0.93	0.81	1.07	0.3290
South	0.71	0.63	0.81	< 0.001
West	0.64	0.55	0.74	< 0.001

## Discussion

In this study, we evaluated the trends and outcomes of hospitalizations due to HCC over 10 years from 2008 through 2017. Our study showed an overall decrease in hospitalizations for which HCC was the primary diagnosis and a decrease in inpatient mortality. There was no significant change in the proportion of HCC hospitalizations discharged to facility. Further, the present study revealed several demographic and hospital-level characteristics and comorbid conditions associated with increased odds of mortality. These findings confirm those of previous studies and provide updated trends on HCC hospitalizations and in-hospital mortality in the US. 

The numbers of hospitalizations decreased from 16,754 (10.8) in 2008 to 14,715 in 2017 (9.4%). This trend reflects the hospitalization burden due to HCC and has a limited interpretation. However, there are prior studies that estimated the rate of HCC-related hospitalizations. For example, a study by Jinjuvadia et al. showed that there was no significant change in the proportion of all admissions due to a primary diagnosis of HCC between 2002 and 2011 [19]. In the aforementioned study, the increase was particularly noted among patients with HCC as a secondary diagnosis and likely reflected in an increase in admissions from underlying cirrhosis and end-stage liver disease-related complications such as hepatic encephalopathy, infection or sepsis, and acute renal failure [34]. A similar retrospective analysis by Kim et al. using the NIS demonstrated that the HCC hospitalization rate increased from 13.6 to 22.1 per 100,000 between 2004 and 2015 [24]. This study included both HCC as a primary and secondary diagnosis in the analysis. Another retrospective study based on data from the US National Cancer Institute’s Surveillance, Epidemiology and End Results (SEER) program that included 13 cancer registries representing 14% of the US population showed that the incidence of HCC increased from 4.1 to 7.7 per 100,000 adults between 1992 and 2015 [10]. Indeed, the incidence of HCC in the US is projected to increase through calendar 2030 [2]. The divergence between our finding of decreased hospitalizations due to HCC against the backdrop of increasing incidence of HCC is intriguing but not surprising. Hospitalization is not necessarily required for the diagnosis since most cases of HCC are diagnosed based on imaging studies, which can be done in the outpatient setting [1]. Another plausible explanation for the decrease in hospitalizations may be increased screening and surveillance for HCC with the increasing calendar year, which allows for potential earlier diagnosis of HCC and a higher probability of benefiting from curative treatment [14]. A recent large study from 12 states in the US demonstrated that the proportion of HCC cases that were local stage increased from 27.8% in 1987 to 54.3% in 2017. Early diagnosis of HCC allows for earlier treatment with favorable outcomes, which translates into reduced complications associated with HCC and hence decreased hospitalizations. As a bridge to transplant or as palliation in advanced stages of HCC, transarterial chemoembolization (TACE) can be performed and recent single-center studies suggest that these patients can safely be discharged home after the procedure without the need for overnight hospitalization [35-38]. Thus, there may have been a trend towards performing this procedure on an outpatient basis in the latter part of the study period, thereby leading to the significant decline in the HCC hospitalization rate observed in this study. The decline in HCC hospitalization is reassuring and has the potential to decrease the economic burden of HCC on the US healthcare system. Further surveillance is needed to ascertain if this trend continues. 

Our finding of a significant decrease in in-hospital mortality among hospitalizations with a primary diagnosis of HCC comports with those of prior studies which demonstrated a downward trend in the in-hospital mortality associated with HCC between 2002 and 2014 [19-21]. This could be attributed to advances in the diagnosis and treatment of HCC which includes but is not limited to liver resection, local ablation therapy, liver transplantation, TACE, hepatic arterial infusion chemotherapy, and systemic therapies with agents such as sorafenib [1]. Increased screening and surveillance for HCC in at-risk patients probably led to the increase in local stage disease at the time of diagnosis between 1987 and 2017 [3]. Early diagnosis of local stage disease potentially allowed for early interventions such as resection and local ablation therapy, and liver transplant. The HCC-specific mortality rate among cases with the localized disease in the study by Mahale et al declined across calendar years of the study regardless of whether the liver transplant was performed or not. Further, the proportion of HCC patients who received a liver transplant within 5 years of diagnosis increased from related liver transplants increased significantly from 0.6% in 1987 to 12.4% in 2013 [3]. Taken together, these suggest that adherence to screening and surveillance guidelines in high-risk patients coupled with early diagnosis, and treatment can further decrease the mortality associated with HCC. 

Our examination of the predictors of mortality among HCC hospitalizations builds on the prior work of others that African American or Black race, [39-42] increasing age, [20,43,44] renal failure, [43] and coagulopathy [43] are associated with increased odds of in-hospital mortality. It is pertinent to highlight that racial and ethnic disparity exists in the quality of healthcare in general and specifically in cancers [45-50]. Blacks are disproportionately diagnosed with advanced stages of HCC, are less likely to receive curative treatment, and are less likely to receive liver transplants [42,51,52]. Additionally, our findings indicate that other comorbidities such as infections (sepsis and pneumonia) and pulmonary circulatory disorders were also associated with increased mortality. This suggests that comprehensive and early multidisciplinary evaluation of HCC patients by other subspecialists such as Infectious diseases has the potential to decrease mortality. 

Patients who discharge to facilities such as SNF are typically older, more medically complex, and have higher hospital readmission rates than those who are strong enough to discharge home [31,53,54]. Among HCC hospitalizations, we found that discharge to facility was associated with increasing age, paralysis, psychosis, and neurological diseases. Similar findings have been documented among hospitalizations with colon cancer [54]. Although there was no significant trend in the HCC hospitalizations discharged to facility, cancer patients discharged to facility have poor outcomes. For example, the mortality within six months was 56% among patients with colorectal, pancreatic, bladder, or lung cancer discharged to facility compared with 36% discharged home [31]. Readmission within 30 days from facility among Medicare beneficiaries is also higher [32,55]. The outcomes of patients discharged to facility need further investigation since there is a paucity of data related to this. 

The present study has several strengths. The NIS is the largest healthcare database in the US representing >97% of the inpatient population when weighted. Thus, the findings from this study are nationally representative. Second, the study provides updated trends on HCC hospitalizations and in-hospital mortality, both of which are important to clinicians, administrators, and policymakers in the evaluation of preventative measures and the allocation of healthcare resources. Third, we examined the predictors of discharge to SNF, which have hitherto not been evaluated in prior studies. Undertaking a study from the NIS database comes with its limitations. NIS data has been maintained since 1988, underwent modifications in 2012, which resulted in inconsistencies in data [56]. This database lacks the feature of “present during admission”. The co-morbidities identified in the present study may not have necessarily been present during the admission of the patients. Thus, the distinction between comorbidities and complications during or post-hospitalization could not be made [57]. Finally, although we believe that the NIS data that we used provided a representative cross-sectional sample of HCC in the US, healthcare in other sectors such as the Veterans Administration system was not included in the study. Finally, administrative databases are susceptible to coding errors and omissions but the HCUP has instituted robust quality control measures to deal with these [58]. Our study has several limitations. First, our study is a retrospective study that makes it subjectable to selection bias. Secondly, the selection of samples relies on accurate coding practices that could have confounded our results. However, the large sample size and robust quality measures by AHRQ in the creation of this database makes it an acceptable database for epidemiological studies. 

## Conclusions

In this population-based study, we demonstrated decreased rate of hospitalization and in-hospital mortality among adult hospitalizations with a primary diagnosis of HCC. There were several demographic, patient-level, and hospital-level characteristics associated with in-hospital mortality and discharge to facility. Further surveillance is required to determine if these trends continue and future studies should focus on the reasons for the declining HCC hospitalization and in-hospital mortality rates. An understanding of these reasons will allow for the development of interventions and strategies to sustain these trends. 
